# Croup

**Published:** 1871-01

**Authors:** 


### CROUP.

No disease to which our children are subject, is more frightful to behold, or leads to more distressing results than croup. Indeed, the danger of the disease is so well understood and the symptoms so readily recognized that the first, hoarse, hollow, cough, indicative of the approach of the disease, is a signal for the fond parent to hasten to the little one's crib and commence a vigorous treatment for its relief.

If the child be from one to two and a half years old, the croup is apt to be of a spasmodic nature, the result of dentition and consequent irritation of gums, stomach and bowels. To such a child should be administered from one half to a teaspoonful of the *syrup of ipicac*, every quarter of an hour, until free vomiting is produced and the stomach thoroughly emptied. Cloths wrung out in hot water should be applied to the child's breast and throat, as hot as can be borne. Let the cloths be applied every five minutes for the space of half an hour, after which rub the skin thoroughly dry, and anoint the chest, throat and nostrils with warm sweet oil or goose oil. Next wrap the child in a warm blanket and return it to its crib, when, if it breathes easier and is disposed to sleep, no further interference will be necessary. The following evening it should be thoroughly anointed again, for fear of a relapse, which is likely to occur for the following two nights.

But, should not this treatment relieve the child, or should it be older and have a more severe type of the disease, with inflammation of the windpipe, extending upward to the throat, and downward to the lungs, accompanied with great hoarseness, a wheezing, hollow, cough, great restlessness, hot skin, flushed face, choaking, and expectoration of stringy, tough phlegm, which the child finds impossible to expel from its throat; then must more vigorous measures be resorted to. Send at once for your family physician, and while the messenger is in search of him do not be idle. Procure at once, a piece of unslacked lime, the size of two fists. Place it in a basin and pour a pint of hot water upon it. Seat the child on a chair, envelope it in a blanket—head and all—place the basin with the slacking lime upon its lap, and allow the child to inhale the gases arising therefrom. In less than ten minutes you will have the satisfaction of seeing the little one breathe freely, for you will have dissolved the tough phlegm and membrane which obstructs his breathing apparatus, and your physician, when he arrives, will tell you that you have done rightly and perhaps saved the life of your child.

There is nothing equal to the fumes arising from slacking lime, for dissolving the secretions that obstruct the windpipe and throat of a child suffering from croup. Lime should be kept in jars, tightly corked, in every house, where there are small children, at this season of the year. The wretched manner in which we are in the habit of clothing our children during the cold weather, renders them constantly liable to colds and croup. We should always be prepared to meet the disease promptly, and in no manner can it be so thoroughly done as by the treatment above indicated. If parents would dress their children more with regard to health and comfort, than to display a pretty limb and shoe, there would be far less cause for disease among them. Cloaks, scarfs and furs about the shoulders and necks of children, with a pair of thin gaiters and thinner stockings upon feet and limbs, may be a *fashionable* way of dressing them, but it is certainly, a very *uncomfortable* and *unhealthful* one.

